# Psoralen downregulates osteoarthritis chondrocyte inflammation via an estrogen-like effect and attenuates osteoarthritis

**DOI:** 10.18632/aging.204245

**Published:** 2022-08-24

**Authors:** Kui Huang, Bo Wu, Zhuhu Hou, Akhlaq Ahmad, Mushtaq Ahmed, Ayesha Ali Khan, Feng Tian, Fan Cheng, Wei Chu, Ke Deng

**Affiliations:** 1 Departments of Orthopedics, The First Hospital of Yangtze University, Jingzhou, China; 2 Departments of Orthopedics, The Jiangling County People’s Hospital, Jingzhou, China; 3 The Second Affiliated Hospital, Guangdong Provincial Key Laboratory of Allergy and Clinical Immunology, The State Key Laboratory of Respiratory Disease, Guangzhou Medical University, Guangzhou, China; 4 Department of Biotechnology, University of Science and Technology, Bannu, Pakistan; 5 Department of Biochemistry and Molecular Biology, Quaid-i-Azam University, Islamabad, Pakistan

**Keywords:** psoralen, estrogen receptor, estrogen-like effects, osteoarthritis

## Abstract

Estrogen and its receptor play a positive role in the development of osteoarthritis (OA). Psoralen is a plant-derived estrogen analog. This study aimed to verify whether psoralen inhibits OA through an estrogen-like effect. First, human primary chondrocytes in the late stage of OA were extracted to complete collagen type II immunofluorescence staining and cell proliferation experiments. Subsequently, estrogen, psoralen and estrogen receptor antagonists were co-cultured with OA chondrocytes, and RT-PCR was performed to detect the gene expression. A rabbit OA model was subsequently made by anterior cruciate ligament transection (ACLT). They were set as Sham group, OA group and Psoralen group, respectively. The articular cartilage samples were taken after 5 weeks of treatment, and the effect was observed by gross observation, histological staining, micro-CT scanning of subchondral bone. The results of cellular experiments displayed that the cultured cells were positive for collagen II fluorescence staining and 12 μg/mL psoralen was selected as the optimal concentration. In addition, psoralen had effects similar to estrogen, promoting the expression of estrogen tar-get genes CTSD, PGR and TFF1 and decreasing the expression of the inflammation-related gene TNF- α, IL-1β and IL-6. The effect of psoralen was blocked after the use of an estrogen receptor antagonist. Further animal experiments indicated that the psoralen group showed less destruction of cartilage tissue and decreased OASRI scores compared with the OA group. A subchondral bone CT scan demonstrated that psoralen significantly increased subchondral bone mineral density (BMD), trabecular thickness and trabecular number and decreased trabecular separation. In summary, psoralen inhibits the inflammatory production of chondrocytes, which is related to estrogen-like effect, and can be used to attenuate the progression of OA.

## INTRODUCTION

Osteoarthritis (OA), the most common joint disease of the elderly, is pathologically characterized mainly by articular cartilage destruction and intra-articular inflammatory conditions [1]. The incidence of OA in elderly women is higher than that in men, especially in postmenopausal women [2]. Studies have shown that the decline in the level of estrogen in the body is closely related to the occurrence and severity of OA [3]. Studies have indicated that estrogen can reverse the degradation of the extracellular matrix caused by inflammation in chondrocytes [4]. Therefore, we believe that estrogen supplementation can alleviate the progression of OA. However, estrogen may lead to the production of breast cancer [5], limiting its use in the clinic.

In recent years, it has been found that there is a compound in part of plants with estrogen-like activity and fewer adverse effects. This class of compounds is called phytoestrogens [6]. Psoralen is a natural source of phytoestrogens. It has antibacterial [7] and anti-tumor [8] properties, as well as the ability to promote osteogenesis [9], and inhibit inflammation [10]. Studies have shown that psoralen inhibits inflammation in synovial cells, protects chondrocytes, and delays OA in rats [11]. Kang Xu et al. [12] displayed that psoralen promotes chondrocyte glycosaminoglycan (GAG) synthesis, collagen synthesis, and expression of cartilage-specific genes. However, none of the current studies elucidated the inhibition of chondrocyte inflammation by psoralen and the possible mechanisms involved.

Therefore, this study aims to explore the mechanism by which psoralen inhibits the inflammatory response in chondrocytes and its effects on cartilage and subchondral bone in OA. The findings preliminarily suggest that psoralen inhibits the inflammation in chondrocytes by activating estrogen receptors. Further experiments showed that it promoted the regeneration of rabbit OA cartilage as well as the recovery of subchondral bone mass.

**Abstract image:**
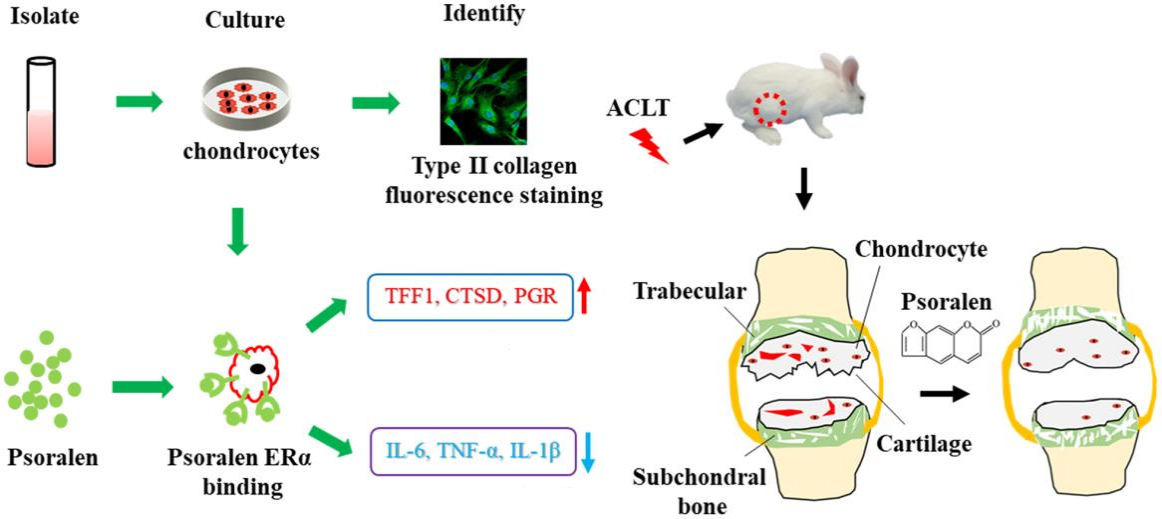
Psoralen inhibits the inflammatory production of chondrocytes, which is related to estrogen-like effect, and can be used to attenuate the progression of OA.

## MATERIALS AND METHODS

### Cell culture and identification

Human primary chondrocytes were derived from cartilage specimens obtained during total knee arthroplasty in patients with OA. The experimental procedures were approved by the ethics committee of the First Affiliated Hospital of Yangtze University, Jingzhou. Briefly, for extraction, the cartilage blocks were first cleaned, sheared and digested with 0.25% trypsin for 30 min at 37° C. Subsequently, the cells were digested with 0.2% collagenase II for 4 h at 37° C and the cell pellets were harvested for culture. The culture medium for chondrocytes was DMEM (Dulbecco’s modified Eagle medium) supplemented with 10% fetal bovine serum (GIBCO). For type 2 collagen fluorescence staining, second passage chondrocytes were harvested, briefly, cultured cells were fixed in 4% paraformaldehyde for 10 min at room temperature, blocked in blocking solution for 1 h. Subsequently, the chondrocytes were incubated with primary antibodies (1:50) against collagen II overnight at 4° C. Next, the chondrocytes were incubated with FITC-conjugated (1:100) goat anti-rabbit secondary antibody for 60 min in the dark. Finally, the chondrocytes were mounted with DAPI (Beyotime Biotech, China) for 5 min. The liquid on the climbing pieces was blotted with absorbent paper and the pieces were blocked with blocking solution containing antifluorescent quencher, and observe and collect the image under a laser scanning confocal microscope (Nikon Japan).

### Cell proliferation experiments

To examine the effects of psoralen on chondrocyte proliferation. Psoralen (Macklin China) was dissolved in a DMSO solution. Second passage chondrocytes were expanded and seeded at a density of 5 × 10^3^/well in 96 well plates and then cultured using different concentrations of psoralen (0 μg/mL, 3 μg/mL, 6 μg/mL, 12 μg/mL, 25 μg/mL) for 1, 3, and 5 days. The cell morphology was examined using an inverted microscope and the proliferation of OA chondrocytes was evaluated using an MTT assay. 100 μL MTT (0.5 mg/mL) solution was added to a 96 well plate and incubated at 37° C for 4 h. Subsequent addition of 100 μL DMSO, wrapped in tin foil paper and placed on a shaker at 37° C for 15 min. Finally, the absorbance was measured at 490 nm using a microplate reader (iMark, Bio-Rad).

### RT-PCR assay

Second passage OA chondrocytes were incubated with estradiol (E2, Aladdin, China) 10^-8^ μM, psoralen 12 μg/mL, estrogen receptor antagonist (fulvestrant, ICI182780) (Beyotime Biotech, China) 1 μM, Estradiol 10^-8^ M + estrogen receptor antagonist 1 μM, Psoralen 12 μg/mL + estrogen receptor antagonist 1 μM. After the cells were cultured for 5d, real-time quantitative polymerase chain reaction (PCR) was performed according to the manufacturer’s instructions using the manual. In brief, the total RNA was firstly extracted and the RNA concentration was further examined, and the nanodrop2000 software was applied for the assay. Subsequently, 1.0 μg/μL total RNA to synthesize cDNA by the operation instructions of reverse transcription kit (TAKARA). The setup program was to perform reverse transcription at 37° C for 15 min, followed by termination of the reaction at 85° C for 5 s. Finally, the DNA amplification process was performed using a Step one Plus real-time PCR system (Applied Biosystems). The required primers included GAPDH, TFF1, CTSD, PGR, TNF α, IL-1 β, IL-6. The amount of relative expression of genes was calculated using 2^-ddct^ and normalized using the internal reference gene GAPDH. Primer sequences are shown in Table 1.

**Table 1:** List of human primer sequences.

**Genes**	**Primer sequences**
GAPDH	F: 5’-GGAGCGAGATCCCTCCAAAAT-3’
	R: 5’-GGCTGTTGTCATACTTCTCATGG-3’
TFF1	F: 5’-CCCCGTGAAAGACAGAATTGT-3’
	R: 5’-GGTGTCGTCGAAACAGCAG-3’
CTSD	F: 5’-TGCTCAAGAACTACATGGACGC-3’
	R: 5’-CGAAGACGACTGTGAAGCACT-3’
PGR	F: 5’-ACCCGCCCTATCTCAACTACC-3’
	R: 5’-AGGACACCATAATGACAGCCT-3’
TNF-α	F: 5’-CCTCTCTCTAATCAGCCCTCTG-3’
	R: 5’-GAGGACCTGGGAGTAGATGAG-3’
IL-1β	F: 5’-ATGATGGCTTATTACAGTGGCAA-3’
	R: 5’-GTCGGAGATTCGTAGCTGGA-3’
IL-6	F: 5’-ACTCACCTCTTCAGAACGAATTG-3’
	R: 5’-CCATCTTTGGAAGGTTCAGGTTG-3’

### Animal experiment design and grouping

The animal experiment was approved and carried out according to the regulations of the Animal Research Institute of Yangtze University, Hubei, China. Twenty-four adult New Zealand rabbits were purchased from the Hubei Research Center of Laboratory Animals. 16 rabbits were randomly selected to establish the OA animal model by anterior cruciate ligament transection (ACLT), same as previously reported methods [13]. Briefly, isoflurane inhalation anesthetic was used to render the animals unconscious, right knee joint skin was disinfected, and the joint capsule were cut open. The anterior cruciate ligament was cut off. This was confirmed by the anterior drawer test. The other 8 rabbits were divided into a sham group by just cutting open joint capsules without ACLT. Antibiotics (penicillin 100000 units per rabbit) were injected subcutaneously once a day for 3 days. One month later, the psoralen group (8 rabbits) was subjected to psoralen (1 mg/mL, 0.1 mL/kg) joint cavity injection [11], and the OA group (8 rabbits) and the sham group were subjected to normal saline (0.1 mL/kg) joint cavity injection once a week, a total of 5 times.

### Histological staining

The cartilage of the knee joint was fixed with 4% paraformaldehyde for 48 hours, decalcified with EDTA for 4 weeks, dehydrated and paraffin-embedded to prepare 5μm thick serial sections. Hematoxylin and eosin (HE) staining, Masson staining and Safranin O/Fast Green staining were performed and then observed under a light microscope. Osteoarthritis Research Society International (OARSI) scores were given by three independent investigators as previously described [14].

### Micro-CT scanning

The subchondral bone of the femoral end of the rabbit knee joint was scanned using micro-CT. The knee joint samples were fixed by 4% paraformaldehyde for 48 h. A high-resolution micro-CT imaging system (Bruker skyscan 1172) was used for scanning (scan thickness 18 μm, voltage 80 kV, current 100 μA). The bone mineral density (BMD) of subchondral cancellous bone was calculated by calibrating the known decay coefficients (250 mg/cm^3^ and 750 mg/cm^3^) of two hydroxyapatite phantoms. Trabecular thickness, trabecular separation, and trabecular number were subsequently quantified.

### Statistical analysis

SPSS19. 0 software for processing the data. The two independent group samples used the t-test, and the multiple group samples used a one-way analysis of variance (ANOVA). P values less than 0.05 were considered significant.

## RESULTS

### Chondrocyte type II collagen immunofluorescence and proliferation

**Figure 1:**
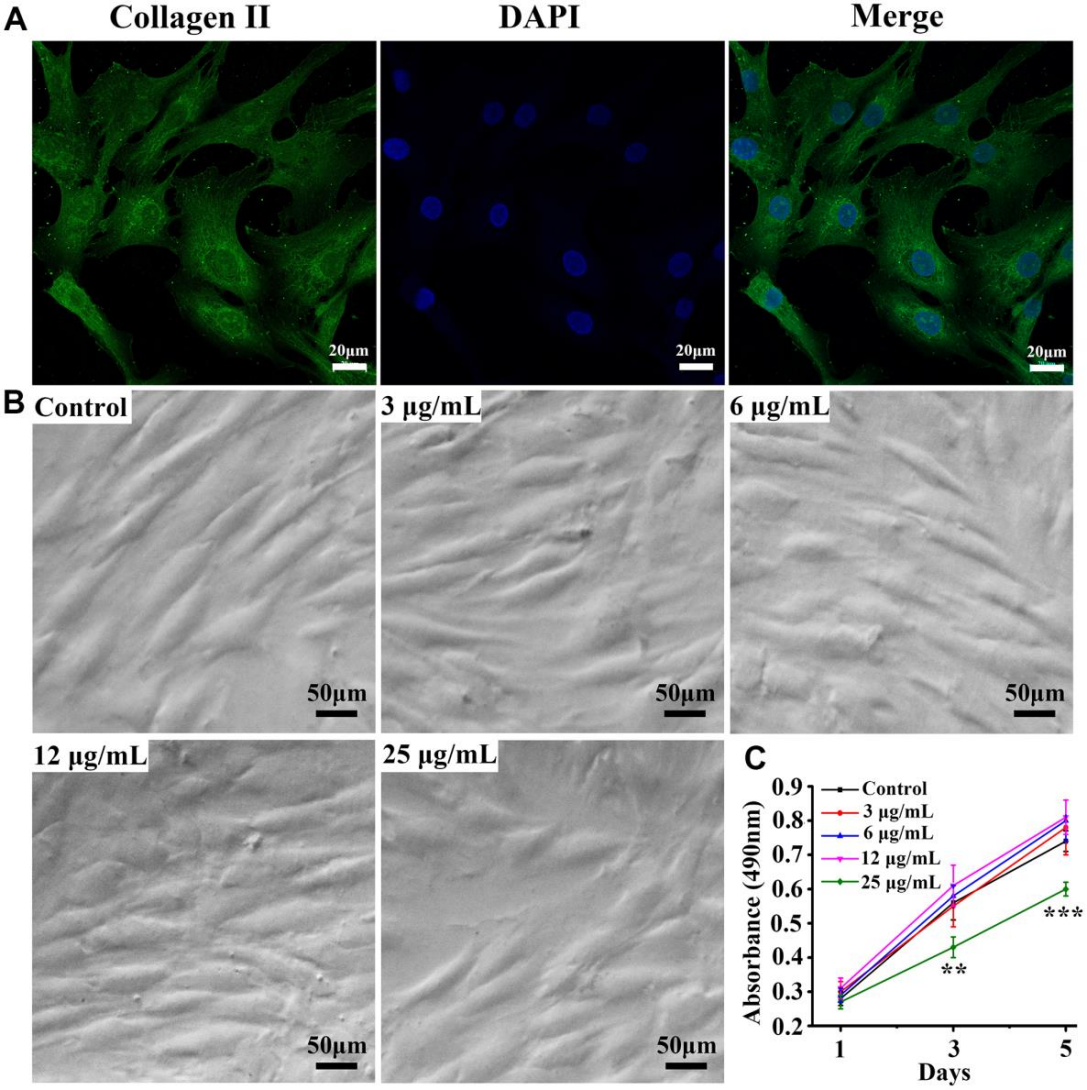
**Identification of chondrocytes and proliferation of cells at different concentrations.** (**A**) Chondrocyte collagen II immunofluorescence staining, (**B**) cell proliferation under culture conditions with different concentrations of psoralen, (**C**) MTT quantitative analysis of the number of cells on days 1, 3, and 5. ** represents P < 0.01, *** represents P < 0.001 compared to control group.

Whether the extracted primary cells were chondrocytes, type II collagen immunofluorescence staining was performed. As displayed in Figure 1A, the cultured cells were positive for collagen II fluorescence staining. Further, we also observed the proliferation of cells in Figure 1B. The morphology and growth of cells were observed on day 5 under an inverted phase-contrast microscope. The cells had a spindled or polygonal shape. At a psoralen concentration equal to 25 μg/mL, the number of cells was significantly reduced as shown in Figure 1C. The proliferation of cells was quantified on days 1, 3 and 5 by MTT assay. When the psoralen concentration was less than 12 μg/mL, it did not affect chondrocyte proliferation. On the other hand, when the concentration equaled 25 μg/mL, it inhibited the proliferation of chondrocytes remarkably, and the difference was statistically significant. Therefore, a dose of 12 μg/mL was selected as the optimal concentration.

### Expression of estrogen target genes

**Figure 2:**
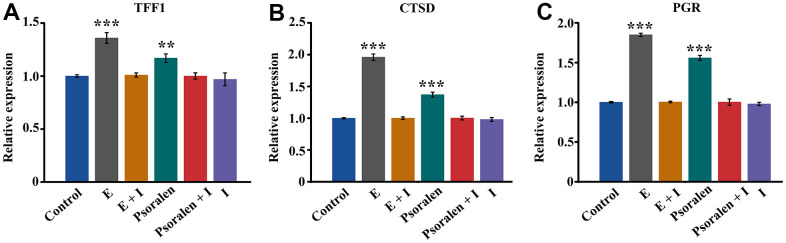
**Expression of estrogen receptor target genes TFF1, CTSD, and PGR.** E represents estrogen and I represents estrogen receptor antagonist. (**A**) Expression of TFF1 gene, (**B**) Expression of CTSD gene, (**C**) Expression of PGR gene. ** represents P < 0.01, *** represents P < 0.001 compared to control group.

Expression of estrogen target genes TFF1, CTSD, PGR was assessed by RT-PCR. As shown in Figure 2A, the expression of TFF1 in the estrogen group was significantly higher than that in the control group, and the difference was statistically significant. The expression of TFF1 in the psoralen group was also higher than that in the control group, and the difference was statistically significant. After the addition of estrogen receptor antagonists, the expression of TFF1 in both estrogen + I group and psoralen + I group compared with the control group did not change significantly. Similar results were obtained as in Figure 2B, 2C. Estrogen and psoralen were able to promote TFF1, CTSD and PGR expression, however, this effect was abolished by the addition of an estrogen receptor antagonist.

### Inflammation related gene expression in OA chondrocytes

**Figure 3:**
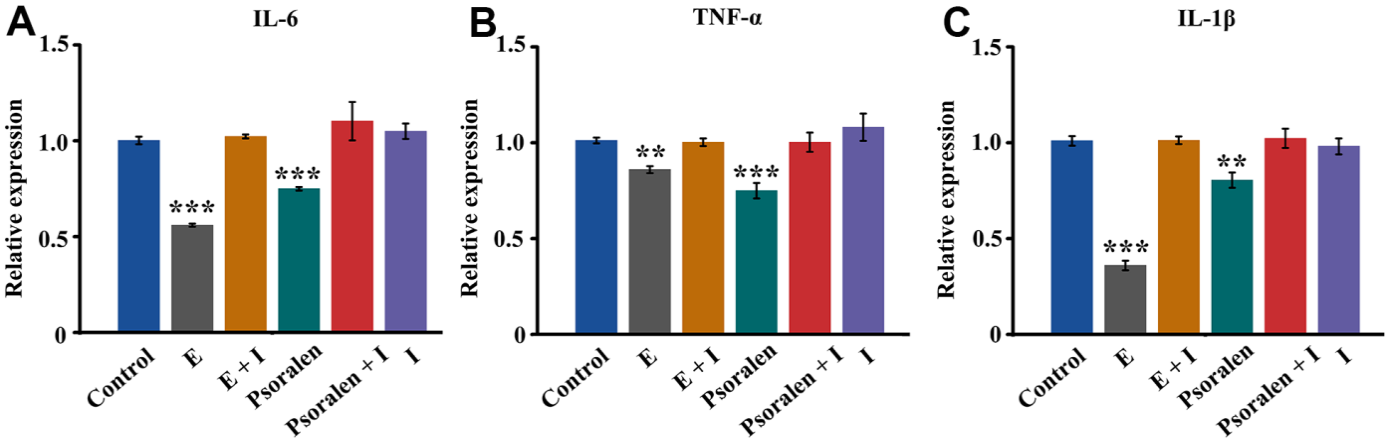
**Effect of psoralen on inflammatory genes IL-6, TNF-α and IL-1β in OA chondrocytes.** E represents estrogen and I represents estrogen receptor antagonist. (**A**) Expression of IL-6 gene, (**B**) Expression of TNF-α gene, (**C**) Expression of IL-1β gene. ** represents P < 0.01, *** represents P < 0.001 compared to control group.

IL-6, TNF-α, IL-1β are inflammation-related genes. It is proved that psoralen can inhibit the inflammation of OA chondrocytes by examining their expression. As displayed in Figure 3A, the amount of IL-6 expression in the estrogen group and the psoralen group was significantly lower than that in the control group, and the difference was statistically significant. After the addition of the estrogen receptor antagonist, the expression of IL-6 in the estrogen + I group and psoralen + I group was higher than that in the control group, but the difference was not statistically significant. As shown in Figure 2B, 2C, estrogen and psoralen were able to decrease TNF-α and IL-1β of expression, however, this effect disappeared with the addition of an estrogen receptor antagonist.

### Psoralen favors repair of OA cartilage in rabbits

**Figure 4:**
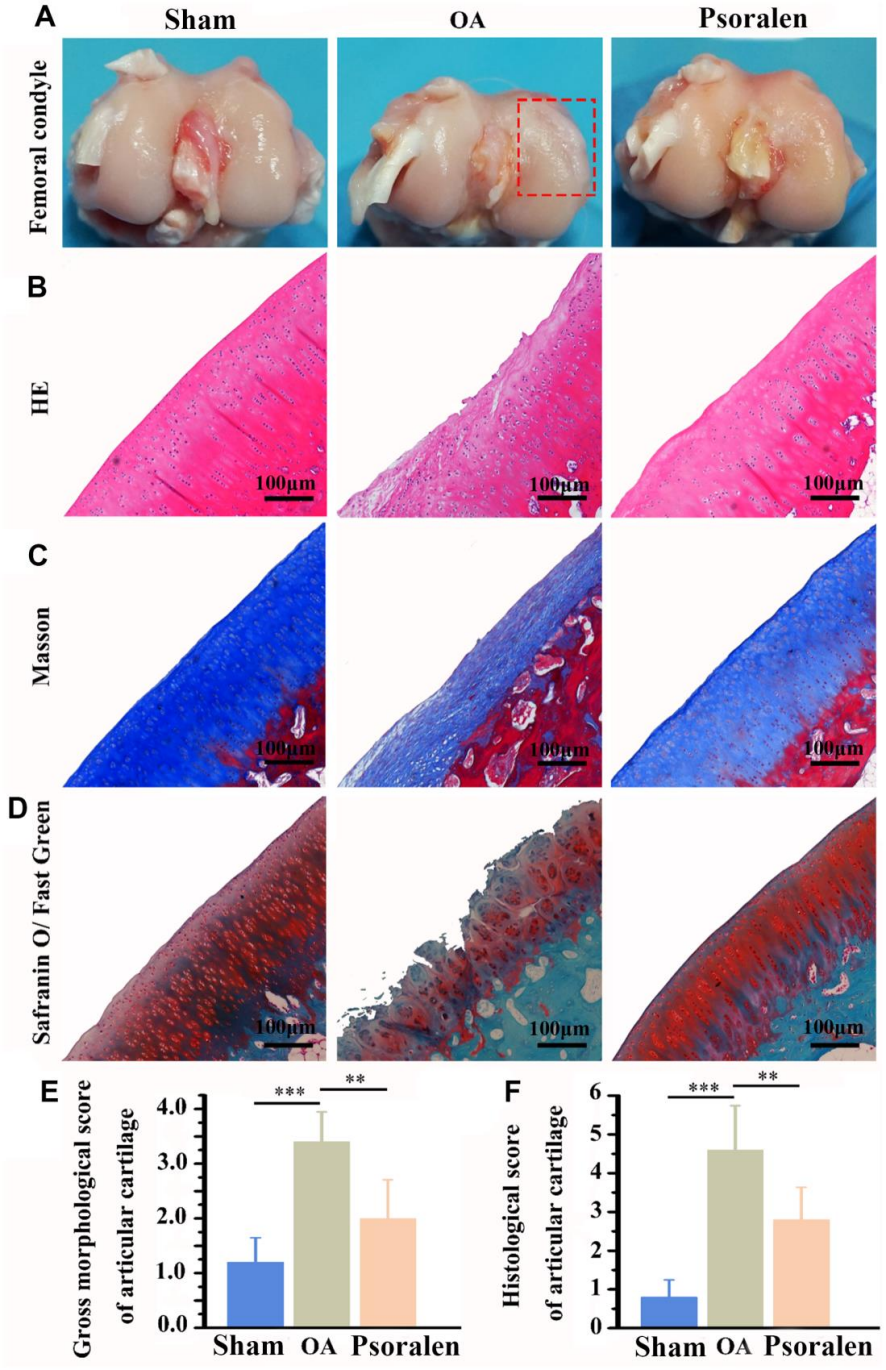
**Intra-articular injection of psoralen significantly promoted OA rabbit model cartilage regeneration *in vivo*.** (**A**) Macroscopic observation of femoral condylar cartilage. (**B**) HE staining, (**C**) Masson staining, (**D**) Safranin O/Fast Green staining, (**E**) Gross morphological score, (**F**) Histological score. The lower score, the smaller the cartilage damage and the better the repair effect. ** represents P < 0.01, *** represents P < 0.001.

In Figure 4A, compared with the sham group, the cartilage surface of the femoral condyle in the OA group is uneven, locally concave and edematous. However, the psoralen group had only a little edema and slight damage to the cartilage surface. As demonstrated in Figure 4B–4D, the cartilage tissue was stained with HE, Masson and Safranine o/fast green respectively. Compared with the sham group, the OA group has obvious cartilage destruction, cartilage reduction and cartilage thickness reduction. The cartilage structure of the psoralen group was better than that of the OA group, cartilage thickness in the psoralen group was similar to the sham group. Further quantitative score statistics displayed that the gross morphological score and the historical score of the psoralen group were lower than that of the OA group (P < 0.01) as shown in Figure 4E, 4F. The results indicated that psoralen promoted cartilage regeneration.

### Psoralen favors the increase of subchondral bone mass in rabbit OA

**Figure 5:**
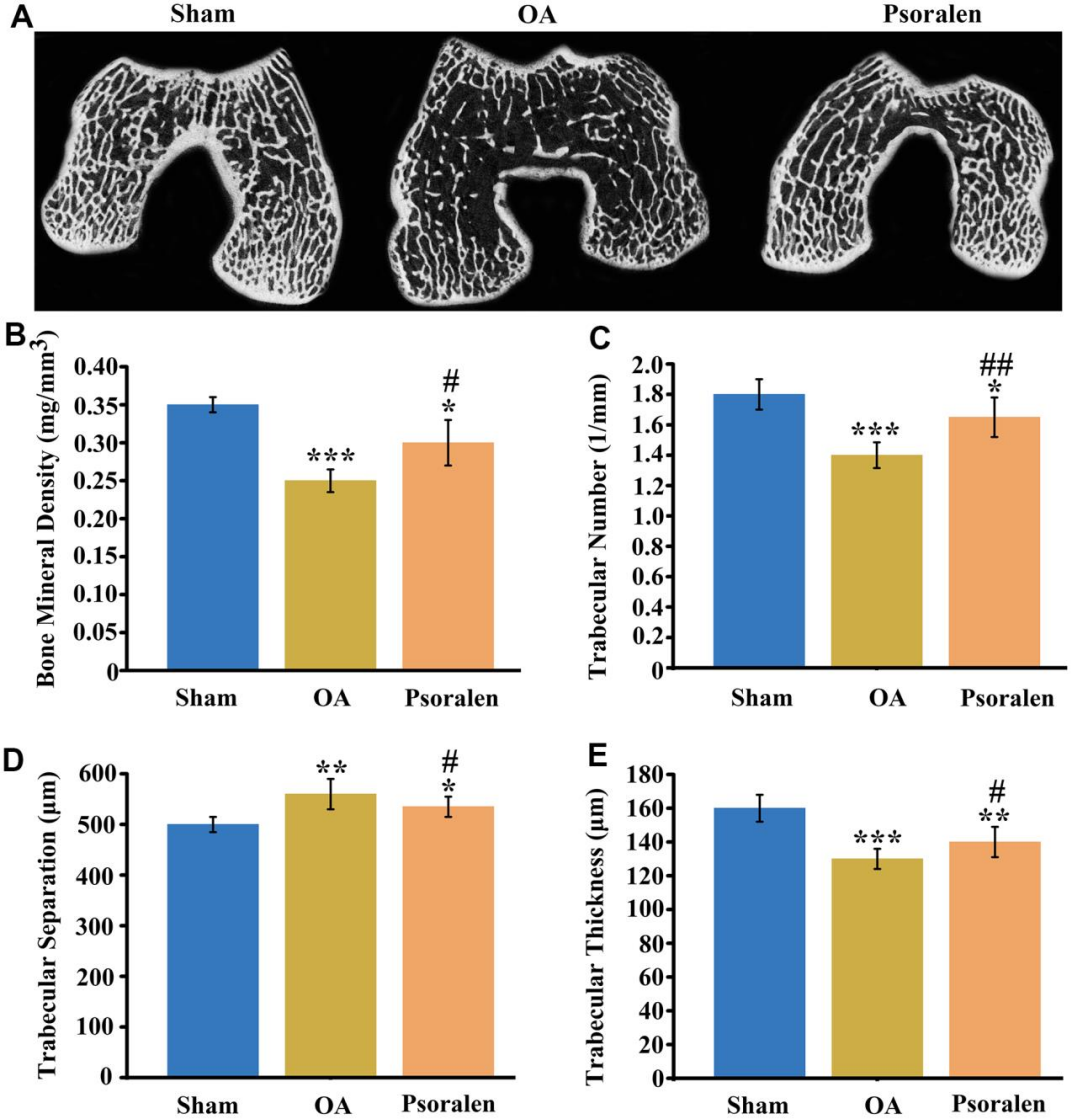
**Psoralen promotes regeneration of bone mass in subchondral bone of rabbit OA.** (**A**) Cross-sectional scan views of the subchondral bone of femoral condyles, (**B**) quantitative statistics of bone density, (**C**) trabecular number, (**D**) trabecular separation, (**E**) trabecular thickness. * represents P < 0.05, ** represents P < 0.01, *** represents P < 0.001 compared to sham group. # represents P < 0.05, ## represents P < 0.01 compared to OA group.

Cross-sectional images of the subchondral bone of femoral condyle were obtained by CT scanning. As shown in Figure 5A, cross-sectional images indicated that the subchondral bone in group OA was significant vacuoles, and the number of trabecular bones was reduced and irregular in comparison with the sham group. The number of trabecular bones increased in the psoralen group compared with the OA group, and no obvious cavity-like changes were observed. Quantitative statistical results of BMD, trabecular number, trabecular separation, trabecular thickness are shown in Figure 5B–5E. The results suggested that BMD, trabecular number, trabecular thickness decreased significantly and trabecular separation increased in the OA and psoralen groups compared with the sham group. However, compared with the OA group, the psoralen group demonstrated significantly higher BMD, trabecular number, trabecular thickness, and decreased trabecular separation.

## DISCUSSION

In this study, we verified that psoralen suppressed the expression of inflammation-related genes in chondrocytes, which is related to estrogen-like effect, and *in vivo* validated that psoralen has the effect of inhibiting OA in rabbits. For the first time, we preliminarily revealed that psoralen acts through estrogen receptors to suppress inflammation in OA chondrocytes.

Type II collagen is mainly produced by chondrocytes [15]. Therefore, to identify whether the extracted primary cells were chondrocytes, we performed type II collagen immunofluorescence staining. The results showed that the cultured cells were chondrocytes with positive type II collagen fluorescence staining. The MTT assay indicated that when the concentration of psoralen was 25 μg/mL, which significantly inhibited chondrocyte proliferation. When the concentration of psoralen was 12 μg/mL or less, it did not affect chondrocyte proliferation. Studies have demonstrated that higher concentrations of psoralen affect Wnt/β-Catenin transcriptional activity, which leads to cell cycle arrest [16]. Therefore, 12 μg/mL or less is a suitable concentration for cultured chondrocytes.

Psoralen is a plant-derived estrogen that acts primarily on estrogen receptors α [10]. Activation of estrogen receptors can promote the expression of downstream target genes TFF1, CTSD, and PGR [17–19]. In our experiments, the expression of estrogen target genes TFF1, CTSD, and PGR were all markedly increased in response to psoralen, and this effect was blocked by estrogen receptor blockers. These results suggest that psoralen has estrogenic-like effects. Subsequently, we further examined the expression of inflammation-related genes in response to psoralen. The results displayed that the expression of inflammation-related genes IL-6 and TNF-α, IL-1β were suppressed by psoralen. And this effect is similarly blocked by estrogen receptor blockers. So the genes TFF1, CTSD, and PGR and inflammation-related genes are both estrogen receptor related and are regulated by the estrogen receptor. Estrogen has anti-inflammatory properties, and one of the key mechanisms of action is the interaction of estrogen receptors with nuclear factors κB (NF-κB), which when inhibited results in NF-κB activated signal transduction [20, 21]. These results again suggested that psoralen had estrogen-like effects and suppressed inflammation-related gene expression of OA chondrocytes via activation of estrogen receptors. IL-6, IL-1β and TNF-α are major proinflammatory cytokines involved in OA progression, and a large body of experimental evidence indicates that blocking IL-6, IL-1β and TNF-α expression can inhibit the degradation of OA cartilage matrix [22]. These results preliminarily illustrate that psoralen has anti-OA effects mainly because of its estrogen-like effects.

*In vivo* results showed that psoralen significantly promoted articular cartilage regeneration, as evidenced by significantly lower OARSI scores compared with the OA group. Wenwei Zheng et al. [23] suggested that psoralen was able to promote chondrocyte type II collagen production. Therefore, combined with *in vitro* cell experiments, the chondroprotective effect of the psoralen group may be related to its inhibition of chondrocyte inflammation and promotion of regeneration of cartilage extracellular matrix. Micro-CT scans of subchondral bone demonstrated that the BMD, the trabecular number and the trabecular thickness were increased and the trabecular separation was decreased in the psoralen group compared with the OA group. In the early stages of OA, subchondral bone volume is lost, manifested by thinning of trabecular bone, increased porosity, and decreased bone density [24]. This experiment is a model of early-stage OA only one month after ACLT [25]. Psoralen inhibits bone resorption by inhibiting the activity of osteoclasts [26, 27]. In addition, psoralen also promotes osteogenic differentiation of BMSCs [28]. Huang K et al. [29] manifested that psoralen can promote bone regeneration by regulating estrogen receptor, therefore, the improved subchondral bone mass in the psoralen group may be related to its promotion of bone regeneration. In the early stage of OA, the inhibition of subchondral bone loss is beneficial to inhibit the progression of OA [30]. Taken together, psoralen has anti-OA effects.

Of course, the present study also has certain deficiencies. We verified the inhibitory effect of psoralen on osteoarthritis *in vivo* and *in vitro*, and detected the expression of estrogen target genes and inflammation related genes. However, we lack further validation at the protein level. Therefore, more research is needed in this area.

## CONCLUSIONS

This experiment preliminarily verified that psoralen has estrogen-like effects, and psoralen attenuates inflammatory gene expression in OA chondrocytes through the action of estrogen receptors. A rabbit OA model was constructed by ACLT, and after treatment with psoralen, the cartilage damage was significantly reduced and the cartilage matrix was increased. Second, in the early phases of OA, psoralen increased subchondral bone mass and normalized bone remodeling.
